# Potential of artificial intelligence in the risk stratification for and early detection of pancreatic cancer

**DOI:** 10.20517/ais.2022.38

**Published:** 2023-03-20

**Authors:** Daniela R. Tovar, Michael H. Rosenthal, Anirban Maitra, Eugene J. Koay

**Affiliations:** 1 Department of Gastrointestinal Radiation Oncology, The University of Texas, Anderson Cancer Center, Houston, TX 77030, USA.; 2 Department of Radiology, Dana Farber Cancer Institute, Boston, MA 02215, USA.; 3 Department of Radiology, The University of Texas, Anderson Cancer Center, Houston, TX 77030, USA.; 4 Department of Gastrointestinal Radiation Oncology, The University of Texas, Anderson Cancer Center, Houston, TX 77030, USA.

**Keywords:** Pancreatic cancer, artificial intelligence, early detection, risk prediction

## Abstract

Pancreatic ductal adenocarcinoma (PDAC) is the third most lethal cancer in the United States, with a 5-year life expectancy of 11%. Most symptoms manifest at an advanced stage of the disease when surgery is no longer appropriate. The dire prognosis of PDAC warrants new strategies to improve the outcomes of patients, and early detection has garnered significant attention. However, early detection of PDAC is most often incidental, emphasizing the importance of developing new early detection screening strategies. Due to the low incidence of the disease in the general population, much of the focus for screening has turned to individuals at high risk of PDAC. This enriches the screening population and balances the risks associated with pancreas interventions. The cancers that are found in these high-risk individuals by MRI and/or EUS screening show favorable 73% 5-year overall survival. Even with the emphasis on screening in enriched high-risk populations, only a minority of incident cancers are detected this way. One strategy to improve early detection outcomes is to integrate artificial intelligence (AI) into biomarker discovery and risk models. This expert review summarizes recent publications that have developed AI algorithms for the applications of risk stratification of PDAC using radiomics and electronic health records. Furthermore, this review illustrates the current uses of radiomics and biomarkers in AI for early detection of PDAC. Finally, various challenges and potential solutions are highlighted regarding the use of AI in medicine for early detection purposes.

## INTRODUCTION

**Figure 1.**
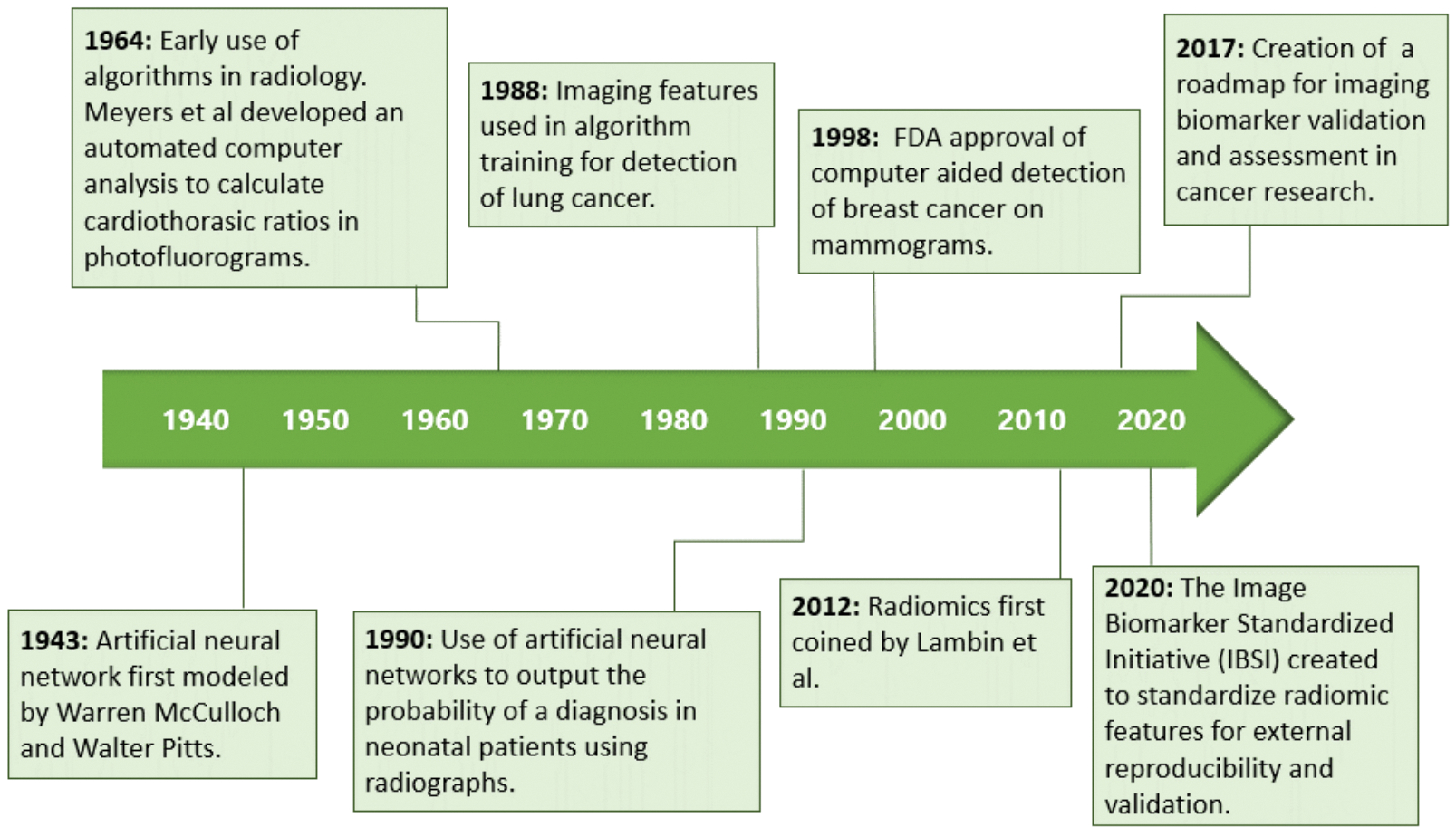
Timeline of the major events and milestones in AI development and its use in diagnostic imaging^[6–13]^.

Pancreatic ductal adenocarcinoma carcinoma (PDAC) is a relatively rare disease, with approximately 62,000 people diagnosed each year in the United States^[1]^. Although PDAC accounts for only 3% of all cancers, it causes 7% of all cancer-related deaths and is projected to become the second leading cause of cancer-related deaths by 2030^[2,3]^. Surgery, in conjunction with chemotherapy (with or without radiation therapy), is the only curative treatment but is appropriate only for 15%−20% of patients^[4]^. Indeed, the high mortality rate of PDAC is attributed to 80%−85% of patients receiving a diagnosis at advanced stages that are not eligible for potentially curative treatments^[5]^. The idea of detecting PDAC at early stages that could be cured using biomarkers and screening methods is an area of intense investigation [Figure 1] and well recognized for the potential to significantly improve the currently dismal 5-year survival rate of 11%^[14]^.

Reports indicate that when PDAC is detected early at a localized stage that is eligible for potentially curative therapies, the 5-year survival rate is as high as 60 to 73%^[15,16]^.

A challenge in detecting PDAC early is the lack of effective screening in the general population. With an estimated incidence of 12.9 cases per 100,000 person-years and low prevalence in the general population, PDAC imposes constraints on traditional metrics of biomarker or model performance for early detection and risk prediction. Key considerations of the performance of any biomarker test or model are positive predictive value, negative predictive value, sensitivity, specificity, accuracy, and area under the receiver operating characteristic curve (AUC). The positive predictive value of a biomarker test poses a particularly daunting challenge for performance with PDAC. For example, while it would seem that a hypothetical diagnostic screening test with 95% sensitivity and 95% specificity for early detection of PDAC in the general population would be desirable, the low incidence of PDAC would lead to an extremely high number of false positive results, giving this hypothetical test a very low positive predictive value of approximately 1.4% (Table 1, adapted from^[5]^ with updated statistics from^[17]^).

Biomarker performance for PDAC screening is especially important considering the potential harms of a definitive diagnosis with tissue. To diagnose PDAC, a biopsy of the pancreas must be done using endoscopic ultrasound with fine needle aspiration (EUS-FNA), biopsy under computed tomography (CT) image guidance, or tissue acquisition from pancreatectomy. The invasiveness of these procedures and their costs remain strong considerations against general screening and any potential benefits of early detection in the general population. Indeed, the US Preventive Services Task Force reaffirmed against screening for PDAC in asymptomatic individuals^[18]^.

To overcome the significant challenge of screening in the general population, researchers have focused surveillance methods for high-risk populations, including patients with multiple first-degree relatives with a history of PDAC diagnosis and high-risk germline mutations, although the frequency and modality (ies) of surveillance of these individuals remains an open research question. Furthermore, another major clinical conundrum is the surveillance of patients who have incidental findings of mucinous cysts such as intraductal papillary mucinous neoplasms (IPMNs) or mucinous cystic neoplasms (MCNs) in the pancreas. Only a small proportion of IPMNs and MCNs undergo malignant transformation, but a high proportion are overdiagnosed and subsequently overtreated^[19,20]^.

In developing new methods to identify PDAC at an early, curable stage, a major focus of PDAC early detection research has been on identifying serum or plasma biomarkers that are specific and sensitive enough for accurate cancer identification. Currently, carbohydrate antigen 19–9 (CA19–9) is the only tumor marker used in monitoring treatment response to chemotherapy^[21]^. However, because of its low sensitivity in the general population, CA19–9 is not recommended as a serum screening test due to the risk of overdiagnosis^[22]^. Multiple other blood-based markers have been described, but none have been adopted as standard clinical tests yet.

Artificial intelligence (AI) is another general strategy that is gaining significant attention for early detection of PDAC and other cancers. Indeed, predictive AI models have been used to assess the risk of developing different malignancies, including oral, breast, and lung cancers^[23–26]^. In these different diseases, AI has been deployed widely, including the use of ANN on health data records and clinical-pathological features^[24]^, development of a CNN model with electronic health records^[25]^, and utilization of ML with imaging^[26]^. In the past four years, there has been a rapid increase in the publications of using AI in pancreatic cancer diagnosis, imaging, treatment, and risk prediction using similar approaches as other disease sites, along with the development of novel methods and algorithms^[27]^.

Broadly speaking, AI techniques include machine learning (ML), convolutional neural networks (CNN), and deep learning (DL), and these methods work by interpreting and analyzing big data [Table 2]. For PDAC early detection, AI has illustrated promise in several domains. One of the principal points of interest involves finding predictors using health records to create predictive models to identify those with a higher risk of developing PDAC. Another focus of AI research investigates the ways that models detect the cancer at a localized, potentially curable stage using biomarkers or imaging.

This expert literature review highlights recent developments of models created to stratify high-risk individuals (HRI) using patient data, including new-onset diabetes mellitus and hyperglycemia, and radiomics, which can identify image features and patient anatomy that are predictive of future malignancy. Also, this review summarizes studies utilizing radiomics for the classification of high-grade IPMNs and tumor detection using CT images, and the classification of cell clusters and microbiota for early detection. Furthermore, the ethical and privacy concerns researchers must consider when training models using patient data, as well as the steps needed to develop a transparent and ethical model that can be clinically adopted, are discussed.

## RISK PREDICTION MODELS

One strategy to reduce the unacceptably high false positive results that stem from the low prevalence of PDAC in the general population is to focus on higher-risk populations. The “sequential sieve” model has been widely adopted for PDAC to enrich screening populations^[33]^. In this model, a first sieve is used to filter the general population to enrich high-risk individuals based on a common phenotype, while a second sieve then filters this enriched cohort to find a blood-based or an imaging-based marker among these high-risk individuals predicted to develop PDAC^[33]^. One of the risk factors is familial risk and germline mutations, representing about 10% of PDAC patients^[34]^. Other risk factors include cystic lesions and new-onset diabetes^[35]^. To better understand the natural history of PDAC in the setting of new-onset diabetes (NOD), a prospective trial is recruiting participants to investigate the incident rate of PDAC in those with new-onset hyperglycemia and diabetes, wherein patients considered to be at the highest risk of harboring an occult (asymptomatic) PDAC will undergo abdominal imaging^[36]^. Another example of a large cohort of patients who are being followed for incident PDAC and treatment outcomes includes the Florida Pancreas Collaborative, which has created biorepositories, including blood, CT scans, and tissue samples from 15 institutions in Florida to address PDAC disparities^[37]^. These cohort-building efforts, aligned with risk prediction models [Table 3], are aimed at improving risk assessment, as well as biomarker discovery and validation.

### Models using health records to assess risk

About 50% of PDAC patients are diagnosed with NOD and 85% are hyperglycemic, both believed to be induced by the tumor^[54]^. Importantly, hyperglycemia can start manifesting on laboratory testing several months to 2 years prior to the clinical appearance of “classic” PDAC-associated symptoms (jaundice, weight loss)^[55]^. In 2017, Boursi *et al.* used health records to create a model to predict PDAC-induced diabetes mellitus amongst a cohort of all new-onset diabetes mellitus patients. Using a study cohort of approximately 180,000 patients with new-onset diabetes mellitus, the trained model analyzed predictors, such as age, smoking, body mass index, as well as blood serum levels within three years of the diabetes diagnosis for stratification (AUC, 0.82)^[38]^. For further stratification, Boursi *et al.* created a model using a study cohort of 138,232 patients with impaired fasting glucose, or prediabetes. The model analyzed those with impaired fasting glucose (IFG), including predictors such as age, body mass index (BMI), and blood serum levels, to train the model to identify high-risk patients (AUC, 0.71)^[39]^.

Using data from nearly 800,000 patients, an ANN was developed by incorporating 18 personal health features from datasets. These variables included data that are ubiquitous in health records, such as the presence of diabetes, race, and family history. The model stratified patients as low-, medium-, and high-risk and performed with an AUC of 0.86^[40]^.

### Image-based risk models

The term “radiomics” refers to a family of image analysis techniques that convert image data into sets of quantitative feature measurements that represent key features like brightness, shape, and texture. The coupling of organ-specific radiomics, which requires organs to be identified within the images for analysis, and AI [Table 2], which can provide both organ labels and integrated analysis of the radiomics features, may be a powerful tool for analyzing routine clinical images alongside a radiologist to provide new clinical insights such as a prediction of malignancy [Figure 1]^[56,57]^.

Several methods utilized in recent research have incorporated image features seen in multiple image modalities, such as computed tomography (CT) and magnetic resonance imaging (MRI), to create models to detect those with a higher chance of malignancy. Qureshi *et al.* conducted a retrospective study of 72 subjects to analyze images to find precursor indicators of PDAC present in pre-diagnostic CT scans, which were taken 3–6 months prior to PDAC diagnosis when indicated as normal by a pathologist. Their Bayes classifier model detected image features able to categorize scans as ‘healthy’ or ‘pre-diagnostic’ with an accuracy of 86%^[41]^.

Chen *et al.* investigated the use of a multi-state model of abnormal pancreatic morphological features from CT and MRI in combination with patient demographics, clinical features, and lab measurements for risk prediction. Out of the PDAC abnormalities evaluated, the most prevalent were pancreatic parenchymal atrophy reported in 21.4% of patients and calcification in 12.6% of patients. Among these morphological features, pancreatic duct dilatation was determined as an additional indicator of PDAC. The model found that those with a calculated risk of more than 5% represented 90% of their total PDAC study population (AUC 0.825–0.833)^[42]^.

## EARLY DETECTION MODELS

### Radiomics-based AI models

Signs of pancreatic cancer have previously been estimated to be detectable 3–36 months before diagnosis^[55]^. Mukherjee *et al.* trained a ML model to detect PDAC at a stage not visible on CT imaging by radiologists. Using a pre-diagnostic cohort of 155 patients and a control cohort of 265 patients, CT scans were manually segmented, then radiomic CT features were extracted and selected. Four ML classifiers were trained, with Support Vector Machine performing the best in classifying CT scans as ‘pre-diagnostic’ or ‘normal’ when evaluating specificity, sensitivity, AUC, and accuracy (AUC, 0.98). All four ML models performed better than the radiologists, who performed with an AUC of 0.66^[43]^. This indicates the promising potential to use AI in conjunction with normal imaging to aid radiologists in detecting potential malignancy. Comparably, another study sought to increase pancreatic detection in tumors smaller than 2 cm, which are often missed by radiologists^[58]^. Using a CNN-trained model, the pancreas and tumor were segmented from contrast-enhanced CT scans. This DL-based computer detection model was used in 546 patients with pancreatic cancer and a control group of 733 in Taiwan^[50]^.

One area of interest is the ability of radiomics to predict malignancy risk in patients with cystic IPMNs, which can transform to PDAC. IPMNs arise from the pancreatic duct and side branches and are estimated to account for approximately 10% of PDAC patients. Notably, 3% of the general population is estimated to have an IPMN, indicating that many of these lesions are benign^[59]^. Predicting whether these neoplasms are malignant on imaging can be a valuable tool in early detection; however, current imaging assessment is challenging and not accurate in predicting malignancy risk. Still, the current Fukuoka International Consensus Guidelines (ICG) (and other guidelines) use morphological imaging features to guide the decision to proceed with surgical resection^[60]^. This carries a risk of overtreatment, since pancreatectomy is associated with the highest rates of morbidity (40%) and mortality (up to 2%) among abdominal surgeries^[61]^. Hanania *et al.* previously showed the correlation between radiomic features and histopathological grade of IPMNs. In their logistic regression model, an AUC of 0.96 was achieved in distinguishing cancer or high-grade dysplasia from low-grade dysplasia in IPMNs^[48]^, demonstrating radiomics-based AI models [Table 3] may be developed as an alternative method of diagnosis that is noninvasive, time efficient, and cost-effective. Similarly, Permuth *et al.* extracted 14 radiomic features for a logistic regression model along with miRNA expression data and clinical factors, resulting in an AUC of 0.93^[44]^. Polk *et al.* built a model to predict IPMN malignancy using CT radiomics. In this multivariable model, both venous and arterial phase scans from patients with histologically confirmed IPMNs were utilized. All scans were separated into two cohorts, “malignant” and “benign”, for model building, with the major image feature differences being pancreatic duct diameter, cyst wall width, and enhancing solid component. Their model achieved an AUC of 0.93 using ICG and radiomic features^[45]^. Similarly, Tobaly *et al.* validated and trained logistic regression models to predict IPMN malignancy using radiomic features, obtaining an AUC of 0.84. Further models were created to predict between the several subtypes of IPMNs, with the best performing model discriminating between the high-grade dysplasia and invasive pancreatic IPMNs (AUC, 0.92)^[46]^.

In addition to CT and MRI imaging, AI models have been applied to endoscopic ultrasound (EUS) images to assess IPMN malignant potential. A study published in 2019 investigated AI usefulness in diagnosing IPMN-associated PDAC using preoperative EUS imaging. Using 3,970 images, the DL algorithm was trained to output the probability of malignancy, performing with an AUC of 0.98 and an accuracy of 0.94. In comparison with human diagnosis accuracy measured as 0.56 at a preoperative stage, the AI model was more accurate^[47]^.

### Detection models

With a specificity of 96% and a sensitivity of 92%, endoscopic ultrasound-guided fine needle aspirations (EUS-FNA) biopsy of solid pancreatic lesions is highly accurate in diagnosing pancreatic cancer using rapid on-site cytopathology evaluation (ROSE)^[62,63]^. Nevertheless, FNA often results in the ambiguous diagnosis of “atypical cells”. In such cases, diagnosis is difficult, and the underlying pathology can be varied, including chronic pancreatitis and benign and malignant lesions^[64]^. To shorten time and effort in detection, AI can assist cytopathologists in diagnosing these difficult cases. Momeni-Boroujeni *et al.* created a multilayer perceptron neural network to better distinguish between benign and malignant cell clusters by segmenting and extracting the cytology features from the 277 images of benign, malignant, and atypical cases. The model performed with an accuracy of 90.6% to categorize the images as benign or malignant when including all three types of cases^[49]^. To increase efficiency and speed of ROSE, Zhang *et al.* used deep convolutional neural network models to segment stained cell clusters and distinguish malignant cells from benign cells. Their cancer identification model performed with an AUC of 0.958 in the internal test and 0.948–0.976 in the external test and achieved a sensitivity of 0.94^[51]^, similar to that of cytopathologists and higher than trained endoscopists^[65]^.

Another area of research has been in the use of the microbiome as a potential early detection biomarker of pancreatic cancer. Bacterial microbiomes within individuals are similar across multiple organs, including the pancreas, duodenum, and oral cavity. Additionally, there is an observable difference in the composition of bacterial species between those with and without pancreatic cancer^[66]^. Kartal *et al.* used shotgun metagenomics and 16S RNA sequencing to distinguish pancreatic cancer cases from controls. Samples were collected from saliva, feces, pancreatic parenchyma, and pancreatic tumor in the Spanish and German cohorts. Although certain bacterial species were found in abundance in the gut in those with PDAC, such as *Veillonella atypica,* other species had reduced in number. Using the 27 species found in the fecal microbiome, they trained a LASSO logistic regression model to distinguish those with and without PDAC with an AUC of 0.84. Notably, no microbiome populations were associated with other clinical variables, suggesting the unique microbiome seen in PDAC patients is due to the tumor growth and is a valid biomarker. With the addition of CA 19–9 serum marker to the model, accuracy in predicting PDAC improved, performing with an AUC of 0.94. Future development of AI and microbiome populations may provide an accessible and noninvasive population-wide method of detecting PDAC during a curable stage^[52]^.

## FUTURE DIRECTIONS AND CLINICAL ADOPTION

### Federated learning

Research collaborations between different institutes can provide more meaningful data for model training, especially when studying rare diseases such as PDAC. The federated learning approach to collaboration involves sending computer models from one institution to another without sending or exchanging patient data^[67]^. In the standard development of models, concerns over patient privacy remain a large barrier to the collaboration and expansion of data sets. Federated learning is beneficial in that the patient’s information stays locally within the institution^[68]^. The use of federated learning in pancreatic cancer early detection remains in a nascent phase. As PDAC is a heterogeneous and relatively rare cancer, utilization of more data that spans institutions and demographics is expected to strengthen the ability of AI to predict the risk of malignancy or detect early, potentially curable stages of disease with wider applicability. Indeed, bias is a significant challenge to overcome with AI model building efforts, including the inclusion of underrepresented minorities, rare conditions, and disadvantaged socioeconomic groups. Some examples of successful federated learning in medical literature include its use in predicting future hospitalizations of patients with heart diseases using EHR and in COVID-19 diagnosis using X-Ray and ultrasound images^[69,70]^. Ongoing efforts through NIH will apply this form of collaboration to PDAC early detection^[71]^.

### Beyond risk stratification: subtyping PDAC biology for personalized screening

Several elements may be implemented in future AI model building to ensure optimum performance, accuracy, and personalization. PDAC is a heterogeneous disease where treatment response, tumor growth rate, and clinical outcomes vary. Thus, having a customized screening plan for each patient would make detection at an early stage more likely. In aggressive subtypes, such as high delta tumors^[72]^, doubling time of tumor growth was observed to be faster than those of the less aggressive low delta subtype. Moreover, in comparison to the patients with low delta tumors at diagnosis, the patients with high delta tumors at diagnosis were associated with higher blood glucose levels in the pre-diagnostic period, faster wasting of muscle and fat, and more advanced, incurable stages at diagnosis. Creating an AI model that predicts whether a patient will have an aggressive or indolent form of the cancer may help form scheduled surveillance better suited to detect signs of malignancy before metastasis^[53]^.

### Clinical application of AI models

Multiple challenges remain with clinical implementation of AI for early detection of PDAC. Awareness of the ethical and privacy concerns involved in examining patient data at population scales is essential to creating a trustworthy model. Privacy underprotection and overprotection of patient information is a major concern when using big data. While underprotecting data can lead to breaches in privacy, overprotecting can inhibit or block innovation^[73]^. In the context of PDAC, new developments that balance data protection concerns are needed as early detection strategies are integrated into health systems. In addition, there are ethical pitfalls in implementing AI models in a healthcare setting. For example, there may be instances when the AI and physician disagree on a diagnosis, where the physician can explain their reasoning in their judgment, whereas AI cannot provide an explanation. Without a clear justification, the patient may not be given enough information to make the best decision for his or her own health. The physician may keep their original diagnosis, but in the case that it is wrong, it will appear as if they were disregarding crucial evidence. They may also be pressured into agreeing with the model, trusting its accuracy more than their clinical judgement^[74]^.

The start of every model building begins with thinking of its purpose and reviewing literature on the appropriate material and current models for the development of a clinically useful model. An appropriate AI algorithm is chosen with consideration of its desired purpose and the maintenance of patient privacy and consent. External evaluation will provide the most accurate analysis of the model’s reproducibility, which is important for further clinical trials^[75]^.

With the growing complexity of AI used and its influence in medicine, there is a need to provide transparent reporting in its trials. In a study evaluating image-based diagnostic AI study design, only 6% of papers examined included external validation in their methodology, an essential component for thorough clinical evaluation^[76]^. The minimum information about clinical artificial intelligence modeling (MI-CLAIM) checklist was intended to provide transparency in the documentation of the development of these algorithms, including an evaluation of bias and instructions for external reproducibility. In each clinical trial, MI-CLAIM starts with describing the study design, where the researcher answers: 1. What will the algorithm be answering, and how would this fit in a real-world scenario? 2. How is the performance measured and how is it used to evaluate its performance in a clinical setting? 3. Is the cohort representative of a real-world population? 4. Is the testing model performing better than the current models?

Next, the MI-CLAIM has the researcher document each step in the model testing and training, highlighting the methods by which groups were separated to ensure the testing model is representative of the clinical population. The model’s type is then selected, describing which were the best parameters found and how the data was picked, cleaned, and formatted. Statistical performance will be listed, as well as clinical performance evaluators, such as specificity and sensitivity. An examination of the model will provide readers and evaluators with information on the model’s performance, reliability, and significance in the field. To implement the AI in the clinical setting, the researcher’s ultimate goal, the code, computer requirements, notes, or any factors needed for the model building are provided or externally evaluated for reproducibility and accuracy^[77]^.

**Figure 2.**
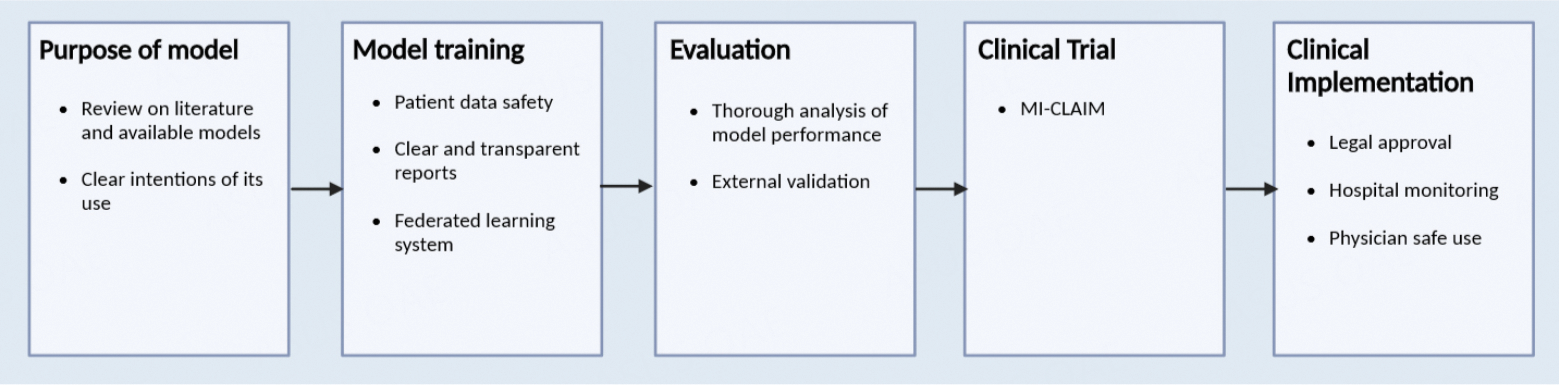
Summary of the development and implementation of AI in medicine^[67,73,75,77]^.

After conducting the clinical study, each model must receive approval from the governing health institution for its clinical adoption. The Food and Drug Administration (FDA) is the governing institution in the United States regulating the clinical implementation of medical technology and treatments. Furthermore, AI models utilized in hospitals need to be monitored and regulated in their practice, considering the ethical and privacy concerns involved, including the requirement of patient consent. Physicians can consider its use and how much influence the AI will have in decision-making [Figure 2]^[75]^.

## CONCLUSION

This review summarizes the recent developments in which AI has the potential to aid early detection efforts. Risk prediction models have been developed by focusing on factors associated with PDAC, such as new-onset diabetes, to identify those who may benefit from surveillance imaging. With proper validation and development, AI may be used as an aid for clinicians to detect cancer growth at a curable stage by using blood-based markers, radiomics and analyzing fecal microbiome composition. In the development of AI models, ethical and privacy concerns should be carefully addressed before full implementation, including data protection and discordant conclusions between AI and physicians. Future studies incorporating federated learning may advance these efforts by assembling large and diverse data while ensuring patient data privacy. In building AI models for clinical implementation, considerations of transparency about the model application and in what settings AI should be deployed are critical to ensure proper use for PDAC early detection and other AI applications.

## Figures and Tables

**Table 1.** Hypothetical results of a biomarker screening test of the general population with sensitivity of 95% and specificity of 95%, giving a PPV of 1.4%

	Patients with PDAC	Patients without PDAC

Positive biomarker test	40,159 (95%)	2,797,502 (5%)
Negative biomarker test	2,114 (5%)	53,152,535 (95%)
All patients	42,273	55,950,037

This hypothetical scenario assumes a U.S. population of 333,287,557 people (based on U.S. Census Bureau data) and applies the biomarker screening test to 16.8% of the population aged 65 years or older. About 2/3 of patients with PDAC are at least 65 years old, with an average age of diagnosis of 70 years (cancer.org). The American Cancer Society estimates that 64,050 people will be diagnosed with PDAC in 2023. PDAC: Pancreatic ductal adenocarcinoma carcinoma.

**Table 2.** Artificial Intelligence definitions

AI	ML	ANN	DL	CNN

A family of computational methods designed to mimic human intelligence and decision-making	Subset of AI that learns to convert input data into a desired output based on analysis of training data^[28]^. Common ML methods include support vector machines, decision trees, and Bayesian networks	Subset of ML designed to mimic biological neural networks. ANNs include computational neurons composed of an input layer, at least one hidden layer, and an output layer^[29]^	Subset of ANNs that contain multiple neural layers between the input and output layers. These networks may contain billions of parameters (e.g, GPT-3 at 175B^[30]^) that form complex representations of patterns from training datasets^[31]^	A class of ANN that uses mathematical convolution - application of a pattern filter to small fields within the data in a manner similar to the human visual cortex - to interpret imaging or audio data^[32]^. There is overlap between CNN and DL networks

AI: Artificial Intelligence; ANN: artificial neural network; CNN: convolutional neural network; DL: deep learning neural network; ML: machine learning.

**Table 3.** Summary of recent AI models utilized in PDAC research

Authors	Year	Model description	AI algorithm	Results

*Risk prediction:*				
Boursi *et al*.^[38]^	2017	Early-onset diabetes, health data	Multivariable logistic regression	AUC, 0.82
Boursi *et al*.^[39]^	2022	Impaired fasting glucose diagnosis, health data	Multivariable model	AUC, 0.71
Muhammad *et al*.^[40]^	2019	Health data, 18 features	ANN	Training test AUC, 0.86 Test set AUC, 0.85
Qureshi *et al*.^[41]^	2022	Risk prediction using radiomics	Bayes classifier	86% accuracy
Chen *et al*.^[42]^	2020	Pancreatic ductal dilation	Multi-state model	AUC, 0.825–0.833
*Early detection:*				
Mukherjee *et al*.^[43]^	2022	Early detection using radiomics	ML	AUC, 0.98
Permuth *et al*.^[44]^	2016	IPMN classification, CT and miRNA	Logistic regression	AUC, 0.92
Polk *et al*.^[45]^	2020	IPMN classification, CT	Multivariate model	AUC, 0.93
Tobaly *et al*.^[46]^	2020	IPMN classification, CT	Multivariate model	AUC, 0.84
Kuwahara *et al*.^[47]^	2019	IPMN classification, EUS	DL	AUC, 0.98
Hanania *et al*.^[48]^	2016	IPMN classification, CT	Logistic regression	AUC, 0.96
Momeni-Boroujeni *et al*.^[49]^	2017	FNA biopsy malignancy	MNN	Stratification of atypical cases as benign or malignant, 77% accuracy
Chen *et al*.^[50]^	2022	Detection of tumors (< 2cm) using radiomics	CNN	Internal test, AUC 0.96 Test set, AUC 0.95
Zhang *et al*^[51]^	2022	Detection of cancer clusters, EUS- FNA	DCNN	Internal test, AUC 0.958 External test, AUC 0.948–0.976
Kartal *et al*.^[52]^	2022	Fecal microbiome	Classifier	AUC, 0.94
Zaid *et al*.^[53]^	2020	Classification of tumors as high delta or low delta	Logistic regression-based binary classification	AUC, 0.84

ANN: Artificial neural network; CNN: convolutional neural network; CT: computed tomography; DCNN: deep convoluted neural network; DL: deep learning; EUS-FNA: endoscopy ultrasonography-fine-needle aspiration; ML: machine learning; MNN: multilayer perceptron neural network; PDAC: pancreatic ductal adenocarcinoma carcinoma.

## Data Availability

Not applicable.
